# Microbial and potentially toxic elements risk assessment in high Andean river water based on Monte Carlo simulation, Peru

**DOI:** 10.1038/s41598-023-48853-4

**Published:** 2023-12-06

**Authors:** María Custodio, Richard Peñaloza, Salomé Ochoa, Heidi De la Cruz, Ciro Rodríguez, Walter Cuadrado

**Affiliations:** 1 Centro de Investigación en Medicina de Altura y Medio Ambiente, Facultad de Medicina Humana, Universidad Nacional del Centro del Perú, Av. Mariscal Castilla N° 3989-4089, Huancayo, Peru; 2 Facultad de Ingeniería Química, Universidad Nacional del Centro del Perú, Av. Mariscal Castilla N° 3989-4089, Huancayo, Peru; 3 Universidad Nacional Autónoma Altoandina de Tarma, Jr. Huaraz 431, Tarma, Peru

**Keywords:** Microbiology, Ecology, Environmental sciences, Environmental social sciences, Risk factors

## Abstract

The study evaluated microbial and Potentially Toxic Elements—PTEs risks in high Andean river water in Peru using Monte Carlo simulation. A total of 144 water samples were collected from four rivers and evaluated for physicochemical parameters, PTEs and bacterial pathogens. The microbial risk analysis for exposure to pathogens present in the water was based on the probability of occurrence of diseases associated with *Escherichia coli*, *Pseudomonas aeruginosa* and enterococci. PTEs risk analysis was performed using a Monte Carlo simulation approach. The results showed that the highest microbial risk due to exposure to water contaminated by *E. coli*, *P. aeruginosa* and enterococci was recorded in the Miraflores and Chia rivers. Meanwhile, the analysis of carcinogenic and non-carcinogenic risk by PTEs in adults and children revealed that the Chia river presents a high risk of contamination by PTEs, especially the carcinogenic risk for children. The Monte Carlo simulation indicated a 56.16% and 94.85% probability of exceeding the limit value of 0.0001 for carcinogenic risk in adults and children, respectively. It can be concluded that children consuming the waters of the Chia river are potentially at risk of As toxicity.

## Introduction

Water quality has a major impact on the health status of consumers. Global water use over the last century has grown at twice the rate of population increase^1^. An estimated 159 million people depend on surface water consumption, 144 million of whom drink untreated surface water^2^. Unsafe water is a threat to public health, putting people at risk for a variety of diseases, including diarrhea, cholera, dysentery, typhoid fever, and polio^3,4^. Globally, water pollution causes about 5.3 million deaths of children under 5 years of age and half of these deaths occur in developing countries^5^. Lack of basic services favors prevalence of waterborne diseases^6^.

Water quality is fundamental to human development and well-being, but increasing urbanization, industrialization and agricultural activity have greatly deteriorated surface water quality worldwide^7,8^. The main sources of water pollution are wastewater discharges and runoff from agricultural and urban areas that carry pathogens and PTEs. Although some countries have close to 100% coverage in the collection and treatment of urban wastewater, only about 63% of the total wastewater generated worldwide is collected and 48% is discharged without treatment, which deteriorates the quality of surface water^9,10^.

In developing countries, water quality monitoring is still performed by enumeration of total coliforms, thermotolerant coliforms, *Escherichia coli* and enterococci^11^. Generally, counts of fecal indicator organisms are determined by membrane filtration or multi-tube fermentation. Both procedures have disadvantages, including long incubation times until confirmation, possible interference by heterotrophic plate count bacteria, and difficulties in interpreting the results^12,13^. New methodologies allow easier interpretation of results in a shorter period. One such method is the IDEXX laboratory's Defined Substrate Technology (DST) initially developed for the detection of fecal coliforms and *E. coli*, and later for *Pseudomonas aeruginosa* and enterococci. The use of IDEXX kits also requires minimal space and equipment^8^.

Currently, water treatment practices have reduced the incidence of disease, but waterborne diseases have not been eliminated. Water contamination by infectious agents causes various diseases, such as cholera, enteric fever, hepatitis, and intestinal flaccidity^14^. Unsafe water is a public health threat, which puts people at risk of diarrhea, other diseases, and chemical poisoning^15^. Therefore, to ensure and maintain good health, water must be of good quality and meet national standards and those recommended by the World Health Organization (WHO) and the U.S. Environmental Protection Agency (EPA)^16^.

Contamination of surface water by PTEs due to untreated wastewater discharges is another concern because of the population that makes direct and indirect use of this resource. Effective public health risk management in surface water bodies exposed to contamination requires information on an ongoing basis. Since it is complex to determine the concentration of microbial pathogens under a wide range of conditions, microbial and PTEs risk assessment is important for estimating health risks and formulating water quality management strategies^17,18^. Therefore, the objective of this study was to assess microbial and PTEs risks in high Andean river water based on Monte Carlo simulation, Peru. For this purpose, sampling sites were established in each of the rivers according to the potential sources of contamination with respect to the field observation.

## Materials and methods

### Description of the study area

The study area is in the Mantaro river basin (parallels 10º34′30'' and 13° 35′ 30'' south latitude, meridians 73° 55′ 00'' and 76° 40′ 30'' west longitude), in the central Andes of Peru. The mean air temperature is lowest in June and highest in November. In areas above 4000 masl the temperature is around 4.3 °C, in areas between 3000 and 4000 masl the temperature varies between 8.1 and 10.4 °C and, in areas between 2000 and 3000 masl the temperature varies between 14.6 and 17.4 °C. The climate varies from semi-humid in most of the basin to very humid in the northwestern and central-eastern part of the Mantaro basin^19^. The maximum average total precipitation per year is 1020 mm^20^. Relative humidity varies from 42–54.5% in August to 74.3–86% in February^21^.

**Figure 1:**
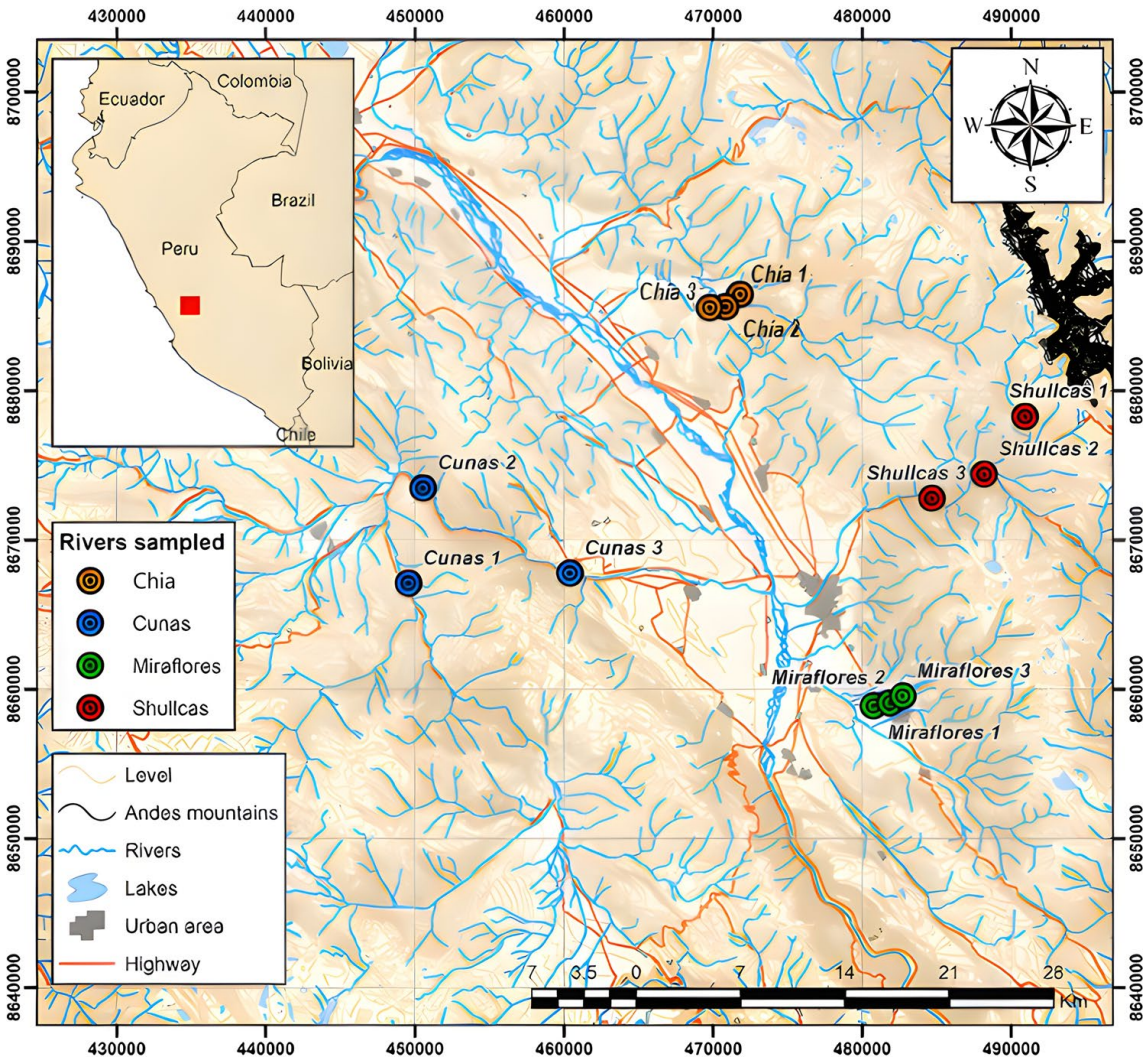
Location map of the study area created using ArcGIS version 10.8^23^.

Three sampling sectors were established in the Cunas, Shullcas, Chia and Miraflores rivers (upper, middle and lower zones) with their respective sampling points (Fig. 1). In the upper zone of the rivers, water is collected for human consumption, in the middle zone for animal drinking, agricultural irrigation and fish farming, and in the lower zone for agricultural irrigation, except for the waters of the lower zone of the Shullcas river (recipient of domestic wastewater). The Cunas river originates in the western mountain range at 5180 masl, flows through the provinces of Chupaca, Concepción and Huancayo in a U-shaped configuration and empties into the Mantaro river at 3220 masl^22^. The Shullcas river is of glacial origin and originates in the Chuspicocha and Lasuntay lagoons, located at the foot of the western flank of the Huaytapallana snow-capped peak. The waters of the Chia and Miraflores rivers are generally used for fish farming.

### Water sampling and analytical determinations

Water sampling was conducted during the dry season (June–August) of 2022 and rainy season (January–March) of 2023, in the sectors established in the Cunas, Shullcas, Chia and Miraflores rivers. A total of 144 water samples were collected in sterile glass bottles 20 cm below the water surface and in countercurrent, in 36 sampling sites (12 sectors), according to the protocol for monitoring the sanitary quality of surface water resources of the General Directorate of Environmental Health of the Ministry of Health^24^. Preservation and transport of water samples to the laboratory for subsequent analysis were performed according to the monitoring protocols of the National Water Authority^25^. At each sampling site, pH, electrical conductivity (EC), total dissolved solids (TDS) (mg/L) and dissolved oxygen (DO) (mg/L) were determined in situ using Hanna Instruments portable equipment (HI 991301 Microprocessor pH/temperature, HI 9835 Microprocessor Conductivity/TDS and HI 9146 Microprocessor dissolved oxygen). Previously, the equipment was calibrated in the respective sampling sector. Water samples for physicochemical determinations were collected in plastic bottles previously treated with hydrochloric acid and rinsed with river water.

EPTs, such as cadmium (Cd), arsenic (As), chromium (Cr) and lead (Pb), were extracted according to the standard method of environmental quality validated by INACAL of Peru (National Institute of Quality), using a mixture of HF, HNO_3_ and concentrated HClO_4_ (5:2:1). The reading was performed with an inductively coupled plasma mass spectrometer (ICP-MS, PerkinElmer NexION 1000)^26,27^.

### Bacteriological analysis

Quantification of *E. coli*, *P. aeruginosa* and enterococci (*Enterococcus faecium* and *Enterococcus faecalis*) was performed using the Defined Substrate Technology (Quanti-Tray system, certified by the U.S. Environmental Protection Agency^28^) according to the manufacturer's instructions. The contents of one sachet of Colilert-18, Pseudalert reagent and Enterolert reagent were dissolved individually in 100 ml of the water sample per test. Each of the solutions obtained were added in 51 wells (QuantiTrayTM) per test, sealed and incubated at different temperatures according to test type (Ramoutar, 2020). Colilert-18 tests were incubated at 35 ± 0.5 °C for 18 h, Pseudalert at 38 ± 0.5 °C, for 24–28 h and Enterolert at 41 ± 0.5 °C for 24 h. After the incubation time had elapsed, fluorescence was searched using ultraviolet light (6 W, 365 nm) and the number of positive wells was quantified to determine the most probable number (MPN) per 100 ml using the IDEXX MPN charts provided (Fig. S1).

### Quality control and quality assurance

Quality control was performed using reference materials and reagent blanks during PTEs analysis. Analytical grade reagents were supplied by Thermo Fisher Scientific (USA) for quality assurance in the procedures. Double deionized water (Milli-Q System, Millipore) was used for the preparation of all solutions. The glassware used was cleaned by immersing it in diluted nitric acid for at least 24 h. They were then rinsed with plenty of deionized water before use. Samples were analyzed in triplicate and the average value was reported. Samples with a variance greater than 5% were discarded and were not used in the evaluation.

### Data analysis

#### Microbial risk assessment

The microbial risk analysis was performed using the quantitative microbial risk assessment (QMRA)^29^. The QMRA included hazard identification, hazard characterization, exposure assessment, and risk characterization that accounts for the probability of disease occurrence and severity of health effects using Monte Carlo simulation. The model calculates a series of iterations to simulate the distribution of outcomes.

#### Human health risk assessment for heavy metals

Human health risk assessment is a quantitative evaluation of the effects on human health resulting from exposure to carcinogenic and non-carcinogenic chemicals^30^. The risk assessment was performed based on the exposure doses to PTEs in water sources via ingestion and dermal exposure using Eqs. (1) and (2).

1 $${ADD}_{ing}=\frac{{C}_{water }x IngR x EF x ED}{BW x AT}$$

2 $${ADD}_{der}=\frac{{C}_{water }x SA x PC x ET x EF x EDxCF}{BW x AT}$$

where D_ing_ is the ingestion dose, D_der_ is the exposure dose through dermal absorption, C is the concentration of PTEs in water, IR is the ingestion rate, EF is the exposure frequency, ED is the exposure duration, BW is the average body weight. AT is average exposure time, SA is exposed skin area, ET is exposure time, CF is unit conversion factor, and PC is dermal permeability coefficient^31,32^. The calculation of carcinogenic risk was performed using Eqs. (3) and (4) as appropriate:

3 $$If \, ADD \leq 0.01, ILCR =(ADD x SF)/L$$

4 $$If \, ADD > 0.01, ILCR =(1 - exp(-ADD x SF)) /L$$

where ADD is the mean daily dose received through ingestion and dermal contact (in mg/kg/day), SF is the slope factor of the PTEs (in mg/kg/day)^−1^ (Hossain et al., 2018), the ILCR value is compared to the acceptable risk level, which is 1 × 10^−4^ according to EPA classification. If the ILCR value is greater than 1 × 10^−4^, the PTEs is considered potentially carcinogenic to the human body, while if it is less than this value the risk is acceptable^33^.

The non-carcinogenic risk has been evaluated through the hazard quotient (HQ), calculated by dividing the exposure value by the reference dose (HQ_ing/der_ is the hazard quotient for ingestion or dermal contact, RfD_ing/der_ is the oral/dermal reference dose)^34^. The overall potential for non-carcinogenic effects has been evaluated by integrating the HQ_s_ calculated for each element and expressed as a hazard index (HI) (HI_ing/der_ is the hazard index for ingestion or dermal contact and "n" is the total number of PTEs studied), Eqs. (5) and (6). The values used for the calculation of the health risk assessment are shown in Tables S1 and S2.

5 $${HQ}_{ing/der}=ADD/\left(Rf{D}_\frac{ing}{der} x L\right)x{10}^{-6}$$

6 $$HI={\sum }_{i=1}^{n}H{Q}_{ing/der}$$

### Monte Carlo simulation

Monte Carlo simulation is a common probability simulation method in risk assessment that can minimize the uncertainty of PTEs concentrations and examine the potential health hazards they pose^30^. In PTEs l health risk assessment, uncertainty is mainly derived from exposure factors, PTEs concentration and the selection of health risk assessment models. This study uses the Monte Carlo method to achieve a true estimate of the health risk value^35^, from the probability of having a cancer risk greater than 1 in 10,000 interactions^18^. The carcinogenic and non-carcinogenic risk values found for the different rivers and the study unit (adults or children) were used. Monte Carlo simulations with an uncertainty analysis were performed with the Excel Risk Simulator extension with an asymptotic probability distribution on the right with the maximum limit value of carcinogenic and non-carcinogenic risk.

### Statistical analysis

All statistical analyses were performed with R software. Spearman's correlation coefficient was used because the data for physicochemical indicators and PTEs were not normally distributed^36^, were coefficient interpreted as 1 indicating perfect positive correlation and -1 indicating perfect negative^37^. Redundancy analysis (RDA) was used to examine the relationship between environmental and microbial variables (Table 1)^38^.

**Table 1:** Environmental quality standards for Peruvian river water^39^.

Physicochemical and biological indicators	A1 Waters that can be made potable with disinfection	A2 Waters that can be treated with conventional treatment conventional treatment	A3 Waters that can be treated with advanced treatment
T (°C)	Δ3	Δ3	**
pH	6.5–8.5	5.5–9.0	5.5–9.0
DO (mg/L)	≥ 6	≥ 5	≥ 4
EC (µS/cm)	1500	1600	**
TDS (mg/L)	1000	1000	1500
TSS (mg/L)			
Cadmium (µg/L)	3	5	10
Arsenic (µg/L)	10	10	150
Chrome (µg/L)	50	50	50
Lead (µg/L)	10	50	50
*Escherichia coli* (NMP/100 mL)	0	**	**
*Pseudomonas aeruginosa* (NMP/100 mL)	**	**	**
Enterococci (NMP/100 mL)	**	**	**

**Not applicable.

## Results and discussion

### Bacteriological analysis of river water collected for human consumption

The concentrations of total coliforms and *E. coli* detected in all rivers exceeded the levels of the national water quality standards (50 NMP/100 mL and 0 NMP/100 mL, respectively). The concentrations of enterococci and the opportunistic pathogen *P. aeruginosa* were similar. Samples collected in the lower sector of the Shullcas river showed very high concentrations of bacteria indicative of fecal contamination and opportunistic pathogens, revealing the strong anthropogenic pressure experienced in this sector of the river. The lower sector of the Shullcas river receives untreated domestic wastewater from the population located along the course of the river in the metropolitan area of the city of Huancayo. The mean concentration of *E. coli* ranged from 28.17 ± 18.33 NMP/100 mL (Shullcas river) to 80.8 ± 65.9 NMP/100 mL (Miraflores river). The mean concentrations of *P. aeruginosa* and enterococci were generally lower in the rivers studied, except in the Chia river (130.8 ± 64.6 NMP/100 mL and 337 ± 457, respectively). Similar occurrence of both microorganisms was recorded in the Cunas and Shullcas rivers. Although total coliforms are not the best indicators of fecal contamination of a water body^40^, high concentrations could indicate a greater health risk when in contact with contaminated water. Some studies report a high correlation between total coliforms and *P. aeruginosa*^41–43^, however, the prevalence and concentration of *P. aeruginosa* in water varies from one water source to another^44^. *P. aeruginosa* is very versatile and can adapt to a wide range of habitats. This adaptability accounts for its constant presence in the environment^45^ and makes it a potential opportunistic pathogen causing a variety of infections from skin rashes to pneumonia^46^.

### Monte Carlo simulation based probabilistic risk assessment for microbial

The analysis of microbial risk due to exposure to pathogens present in the water in different sectors of the rivers studied is based on the probability of occurrence of diseases associated with *E. coli*, *P. aeruginosa* and enterococci and the severity of the effects on health in an exposed population. According to the results obtained in the study, the highest microbial risk due to exposure to water contaminated by *E. coli*, *P. aeruginosa* and enterococci was recorded in the Chia river. The decreasing order of probability of total exposure risk was Chia (5.2 × 10^–1^) > Cunas (2.3 × 10^–1^) > Miraflores (1.8 × 10^–1^) > Shullcas (3.2 × 10^–2^). In the sectors of the rivers where water is collected for human consumption, there is a higher risk of infection by enterococci. In the Chia, Cunas and Miraflores rivers, the risks of infection per 10,000 people exposed per exposure event for *E. coli* and *P. aeruginosa* were lower than the risk for enterococci. In the Chia, Cunas and Miraflores rivers, the risks of infection per 10,000 people exposed per exposure event for *E. coli* and *P. aeruginosa* were lower than the risk for enterococci. The risk of infection for people exposed to the waters of the Shullcas river showed a different behavior compared to the other three rivers evaluated. The risk of infection per 10,000 exposed persons per exposure event for *E. coli* was higher than for *P. aeruginosa* and enterococci (Table 2). It is likely that the combined effect of the microorganisms detected in this study potentiates their action, weakens the human immune system with the consequent presentation of the disease.

**Table 2:** Quantitative microbial risk assessment (QMRA) in river water from the central region of Peru.

River	*E. coli*	*P. aeruginosa*	Enterococos	Total risk
Chia	5.2 × 10^–2^	1.3 × 10^–1^	3.37 × 10^–1^	5.2 × 10^–1^
Cunas	5.1 × 10^–2^	1.0 × 10^–2^	1.73 × 10^–1^	2.3 × 10^–1^
Miraflores	8.0 × 10^–2^	3.1 × 10^–2^	7.1 × 10^–2^	1.8 × 10^–1^
Shullcas	2.8 × 10^–2^	1.8 × 10^–3^	1.8 × 10^–3^	3.2 × 10^–2^

### Analysis of physicochemical parameters and PTEs

Table 3 shows the results of physicochemical parameters, PTEs, microbial indicators, and bacterial pathogens. Temperature values varied according to the river sector, with the highest temperature in the lower sectors of the rivers and the lowest in the upper sectors (near the headwaters). Mean temperature values ranged from 9.15 ± 0.055 °C in the Miraflores river to 11.707 ± 0.821ºC in the Chia river, behavior that would be due to differences in sampling time and ambient temperature. The mean pH values showed an alkaline trend, varying from 7.902 ± 0.455 to 8.336 ± 0.096 in the Cunas river. However, these variations are within the natural ranges for aquatic life, drinking water production and other uses, according to the environmental water quality standards (EWQS) of the Peruvian Ministry of the Environment (6.5–9.0)^47^. As well as, within the ranges established by the World Health Organization (6.5–8.5) (Agency Protection, 2013) and the Canadian Council of Ministers of the Environment (Canadian Council of Ministers of the Environment (6.5–9.0)^48^.

**Table 3:** Mean and standard deviation of physicochemical, potentially toxic elements and bacteriological parameters in river water in the central region of Peru.

Physicochemical and biological indicators	Chia	Cunas	Miraflores	Shullcas
T (°C)	11.707 ± 0.821	10.942 ± 0.488	9.15 ± 0.055	9.85 ± 1.015
pH	8.336 ± 0.096	7.902 ± 0.455	8.0817 ± 0.1665	8.2883 ± 0.1501
DO (mg/l)	7.437 ± 0.404	7.982 ± 0.75	5.578 ± 0.176	6.175 ± 0.524
EC (µS/cm)	355 ± 45.7	377.7 ± 57.4	141.17 ± 5.81	288.17 ± 6.88
TDS (mg/l)	216.7 ± 28.8	238.17 ± 11.25	81 ± 8.29	491.5 ± 17.81
TSS (mg/l)	950.2 ± 101.8	873.8 ± 81	1019.3 ± 120.8	1079.3 ± 163
Cadmium (μg/L)	0.06 ± 0	0.057 ± 0.014	0.06 ± 0	0.17 ± 0.079
Arsenic (μg/L)	13.1 ± 5.32	3.533 ± 0.692	1.45 ± 0.327	5.467 ± 0.989
Chrome (μg/L)	0.817 ± 0.248	1.017 ± 0.183	0.933 ± 0.121	1.017 ± 0.248
Lead (μg/L)	0.148 ± 0.034	1.98 ± 0.018	0.06 ± 0.067	2.25 ± 0.51
*Escherichia coli* (NMP/100 mL)	52.3 ± 26.9	51.83 ± 22.88	80.8 ± 65.9	28.17 ± 18.33
*Pseudomonas aeruginosa* (NMP/100 mL)	130.8 ± 64.6	10.85 ± 9.42	31.3 ± 60	1.833 ± 1.602
Enterococci (NMP/100 mL)	337 ± 457	173.5 ± 31.9	71.8 ± 27.7	1.833 ± 1.602

The mean dissolved oxygen (DO) values ranged from 5.578 ± 0.176 mg/L (Miraflores river) to 7.982 ± 0.75 mg/L (Cunas river). The mean DO values lower than the minimum value of the Peruvian water quality standard (WQS) (6 mg/L) recorded in the rivers studied would be related to wastewater discharges from aquaculture, livestock, and domestic activities. Likewise, these activities could be an important source of microorganisms capable of accelerating the degradation processes of organic matter using the oxygen content of the water^22^. The mean electrical conductivity (EC) values recorded in the four rivers ranged from 141.17 ± 5.81 μS/cm (Miraflores river) to 377.7 ± 57.4 μS/cm (Cunas river) and were lower than those of the ECA (1500 μS/cm). These results are supported by Kükrer and Mutlu^49^ who refer that EC values > 300 μS/cm in water suggest high salinity. However, EC values lower than WQS are an indication of healthy water^50^. The mean values of total dissolved solids (TDS) of the rivers studied exceeded the WQS (≤ 100), except in the middle and upper sectors of the Miraflores river. The variability of EC and TDS observed would be due to the inflow of urban wastewater and runoff from rural areas^51^. High mean values of total suspended solids (TSS) were recorded in the lower and middle sectors of the rivers evaluated.

When comparing the mean values of cadmium (Cd), arsenic (As), chromium (Cr) and lead (Pb) with the national standard values of Peru (10 µg/L)^47^ and the World Health Organization^52^ the mean values recorded were lower than their corresponding water quality standard, except for As recorded in the Chia river. However, these levels may be of concern when considering long-term chronic exposure through water consumption or bioaccumulation and bioamplification in the food chain^53^.

**Figure 2:**
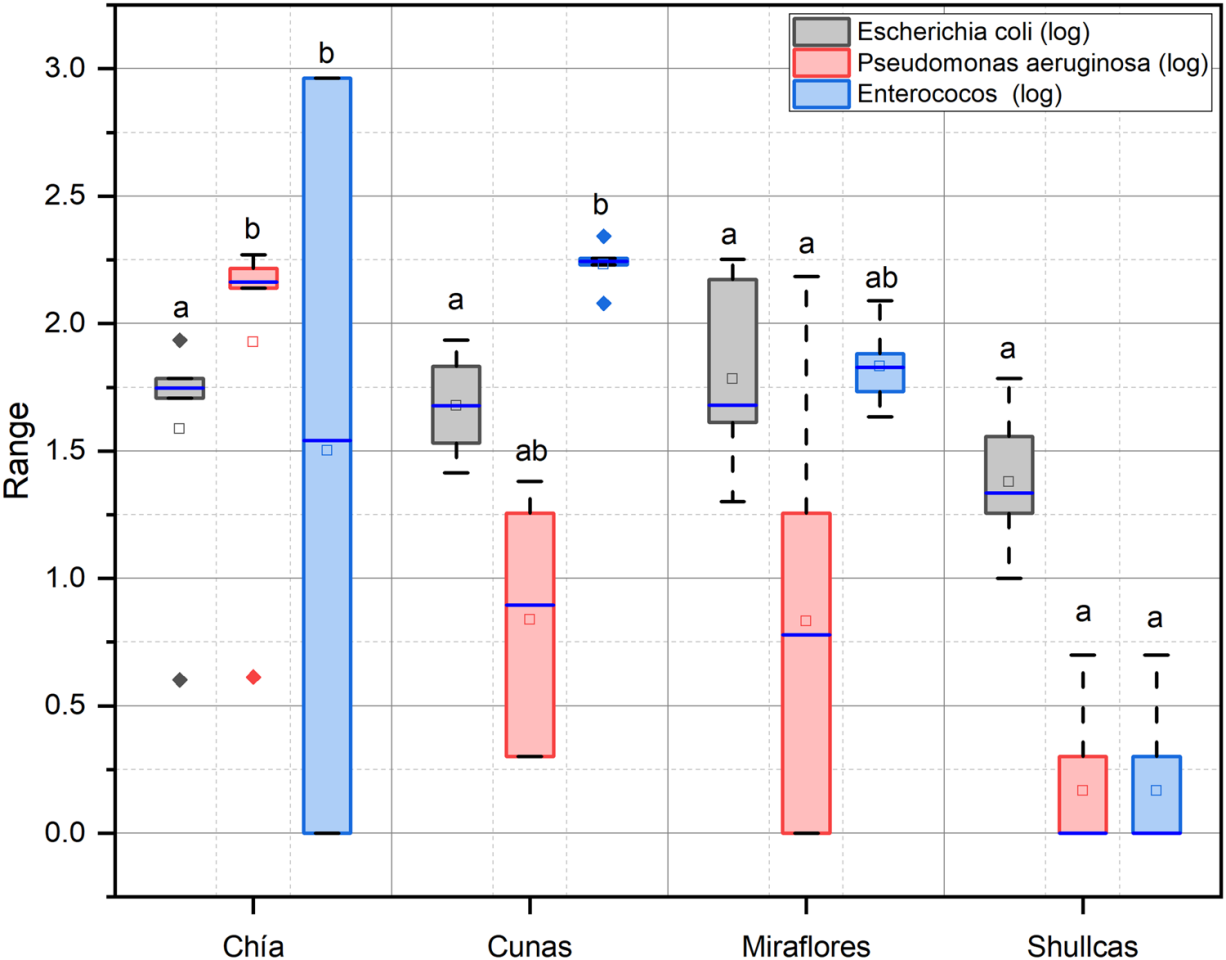
Distribution of bacteria indicating fecal contamination and pathogens by river and sector.

Figure 2 shows the variability of the mean concentrations of *E. coli*, *P. aeruginosa* and enterococci measured in river water in the central region of Peru. In general, high concentrations of microbial indicators were found in all rivers evaluated. Water samples from the Shullcas river had the highest mean concentrations of fecal indicator bacteria and pathogens.

**Figure 3:**
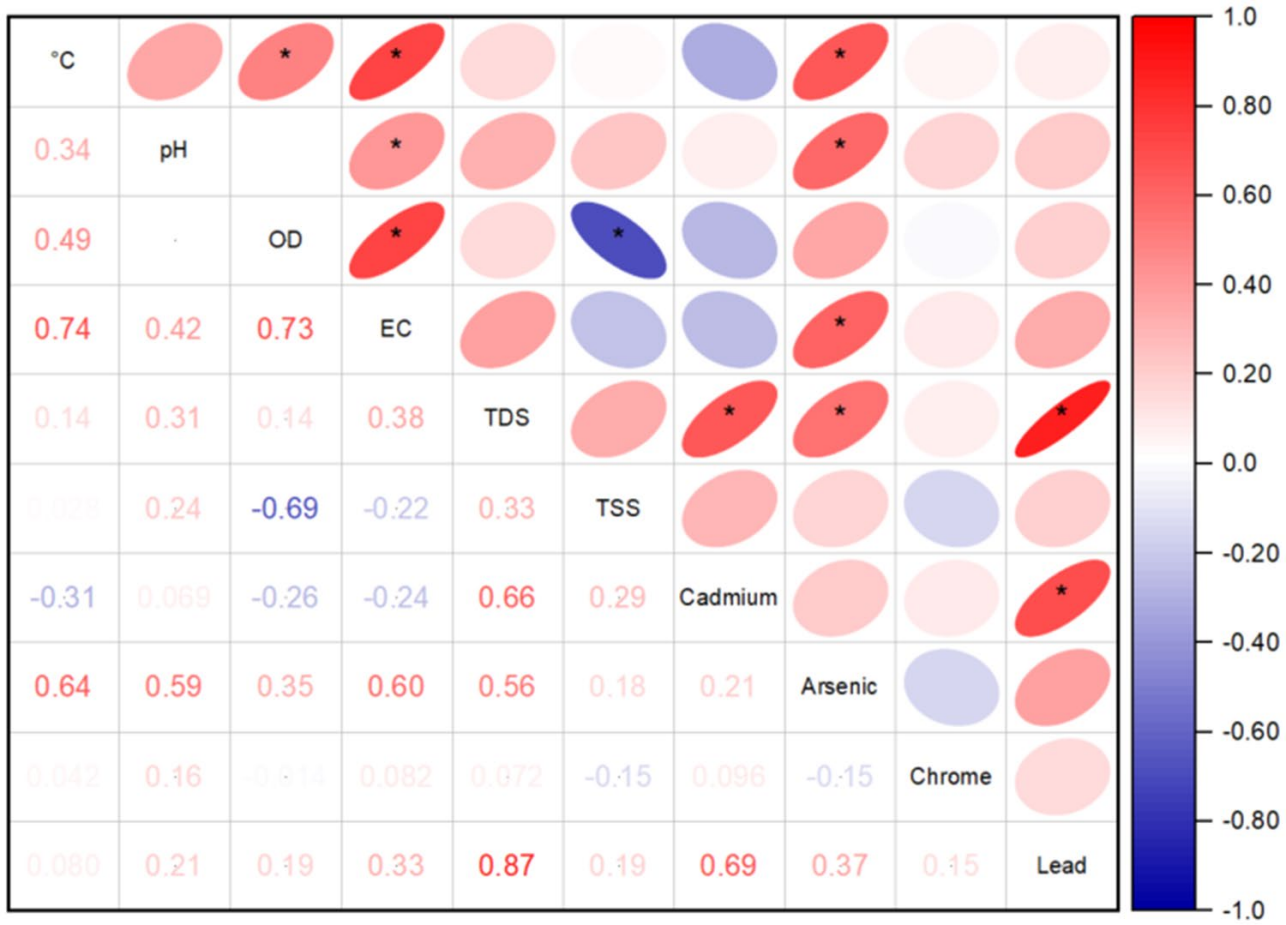
Spearman correlation between physicochemical parameters and potentially toxic elements in river water in the central region of Peru.

Spearman's correlation analysis between physicochemical parameters and PTEs reveals a moderate positive correlation between water temperature (Tw) and DO (r = 0.49) and a strong positive correlation with EC (r = 0.74). These correlations indicate that as water temperature increases, DO and EC tend to increase showing a significant association^54^. This behavior could be due to the amount of salts and minerals dissolved in the water; which, coincides with the findings of Nikolova and Bonev^55^ which indicate that in hard water this relationship is more accentuated. In addition, there is a moderate positive correlation between As and temperature (r = 0.64), suggesting that as As concentration increases, water temperature also increases and this may be due to the solubility and mobility of As in the environment^56^. The correlation of DO with temperature showed a significant positive correlation (r = 0.49), explaining the increase in temperature due to biological activity in the water, which can increase the amount of DO^57^. A positive correlation of DO with EC was also found (r = 0.73). These results are supported by Krishan et al.^58^, who report that EC is associated with the content of dissolved salts and dissolved DO in water. However, a significant negative correlation was reported between TSS and DO (r = − 0.69), indicating that suspended solids in the water may block the pores that exist in the riverbed, which hinders the diffusion of oxygen in the water and decreases the amount that can dissolve^59^. Furthermore, a significant positive correlation was found between Pb and Cd (r = 0.69), it is likely due to the common source of these PTEs, as both can be released by human activity, such as the production of batteries, paints and plastics^60,61^ (Fig. 3).

**Figure 4:**
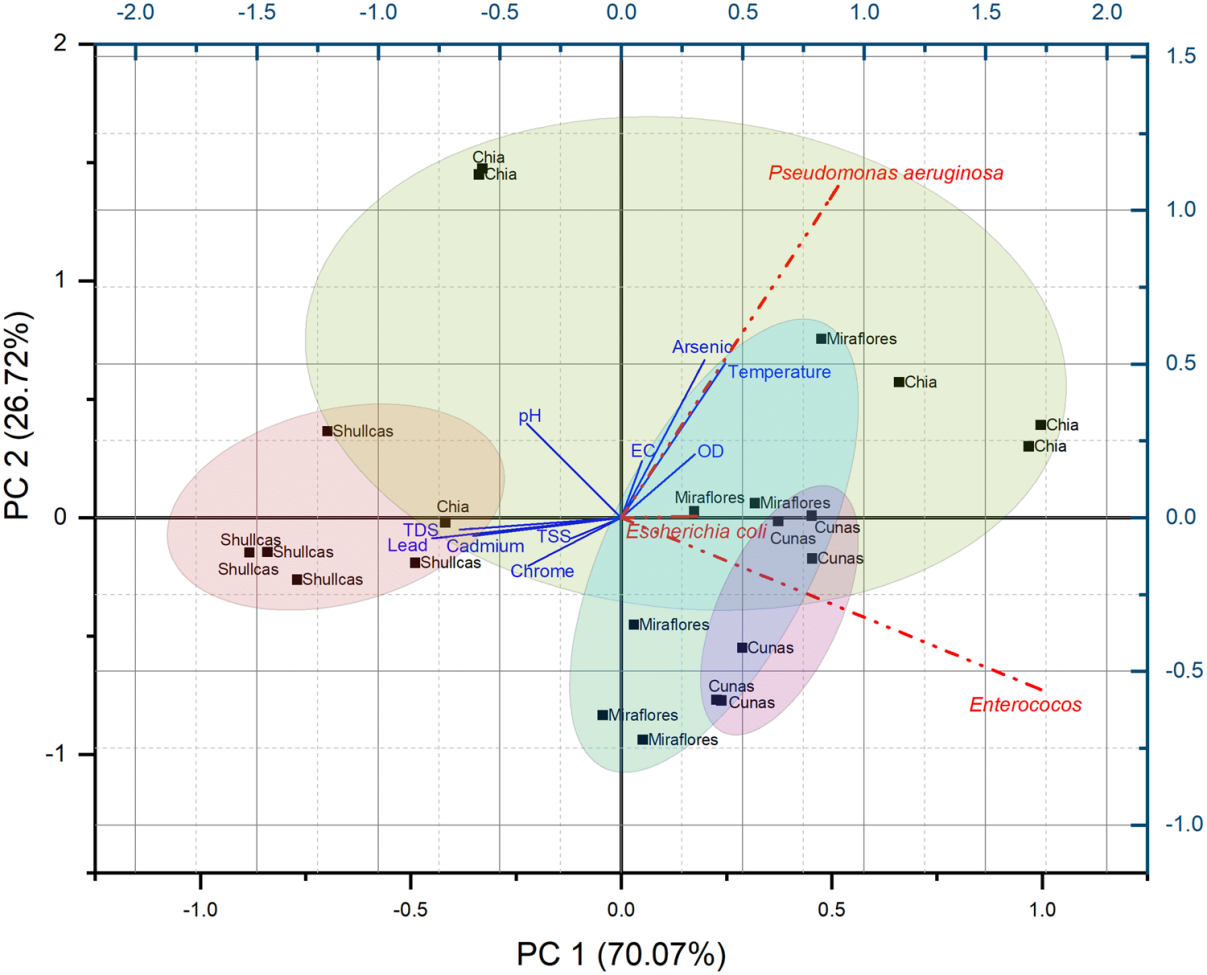
RDA redundancy analysis of physicochemical parameters and potentially toxic elements versus the response of fecal indicator bacteria and pathogens.

Figure 4 and Table S3 show the redundancy analysis (RDA) of physicochemical parameters and PTEs versus the response of fecal contamination indicator bacteria and pathogens detected in river water in the central region of Peru. The coordinates of the response variables on axis 1 reveal the dominance of enterococci (− 0.88), since it has the most negative coordinate on this axis, suggesting that it is inversely related to environmental variables. The first axis explained 58% of the variation and the second axis 22%. On the first axis, the environmental variables that contributed most negatively to the variation were Pb, TDS and Cd with significant loadings, indicating that water quality worsens as the values of these parameters increase. On the other hand, the coordinates of the response variables on axis 2 indicate that the different bacterial species are positively correlated with each other, suggesting that the presence of one bacterial species may indicate the presence of the other bacteria. Enterococci had a negative significant value (− 0.88), denoting that, at higher concentrations of Pb, TDS and Cd, the values of enterococci are reduced. While *P. aeruginosa* with an intermediate negative loading (− 0.45), tends to have a positive correlation of As and temperature, both with low loadings for the first component (− 0.33 and − 0.26, respectively). Therefore, at higher values of these parameters the frequencies of *P. aeruginosa* increase, corroborating that the presence of some bacteria decreases with low water temperatures (below 20 °C)^62^.

In general, water temperature is a critical factor for the growth and survival of fecal indicator bacteria^63^ and the effects may vary depending on the specific temperature and environmental conditions^64^. Another determinant of the distribution of microorganisms in water is the DO. In the RDA analysis, a trend opposite to the other variables was reported, which would correspond to the fact that the higher the frequency of bacteria in the samples, the lower their value, denoting an inverse correlation, corroborating what was reported by Cheng et al.^65^, who found that the reduction of *E. coli* would be explained by an evident increase in pH and DO. Since some coliform bacteria can grow and multiply rapidly in low oxygen conditions^66^. The presence of dissolved oxygen limits the activity of certain enzymes such as glucuronidase that coliform bacteria require for growth making rapid inactivation of *E. coli* and *E. faecalis*^67^.

### Measuring health risk from heavy metals

Table 4 shows the carcinogenic and non-carcinogenic risk values for PTEs for adults and children in water from four rivers. In general, these values for adults are below the 1E−04 limit, suggesting that water consumption in these regions has a low potential risk of causing cancer in adults. However, it is important to note that the Chia river shows a value close to the limit for carcinogenic risk, with a value of 9.80E−05, suggesting that water consumption in this river could have a higher potential risk of causing cancer in adults compared to the risk posed by water consumption in the other rivers studied. Chronic exposure to PTEs through consumption of contaminated water has been associated with a wide range of adverse health effects, including cancer^68^. This type of exposure to PTEs would explain the intermediate frequency of cancer cases reported in central Peru compared to the national estimate^69^.

**Table 4:** Values of health risk of potentially toxic elements for different groups of people in groundwater.

River	Adult		Child	
	Carcinogenic	Non carcinogenic	Carcinogenic	Non carcinogenic
Chia	9.80E−05	4.05E−11	1.43E−04	1.45E−10
Cunas	2.80E−05	6.93E−11	4.08E−05	2.83E−10
Miraflores	1.25E−05	2.10E−11	1.82E−05	8.24E−11
Shullcas	4.26E−05	7.87E−10	6.20E−05	3.30E−09

Chronic exposure to arsenic is implicated in cardiovascular, reproductive, respiratory, neurological, diabetic and gastrointestinal disorders^68,70,71^, especially in vulnerable populations and those living in areas near mining operations^72,73^. Prolonged exposure to cadmium is associated with renal dysfunction, hypertension, anemia, diabetes, osteoporosis and lung diseases^74^. High levels of lead exposure can affect hemoglobin synthesis, renal function, gastrointestinal tract, joints, and the central nervous system^75^. The carcinogenic nature of chromium may be partly explained by the variety of genotoxic lesions it produces, as the mechanism of chromium-associated nephrotoxicity is still unknown^76^.

In children, the carcinogenic and non-carcinogenic risk values for PTEs found are below the 1E−04 limit, except for the Chia river, suggesting that water consumption in these rivers has a low risk of causing cancer. In contrast, the Chia river, which shows a value above the limit for carcinogenic risk, with a value of 1.43E−04, reveals that water consumption in this river could have a higher potential risk of causing cancer in children. Exposure to water contaminated with these PTEs could have important implications for the health of children^77^, as their immune and nervous systems are still developing^78,79^.

### Monte Carlo simulation-based probabilistic assessment for heavy metals

**Figure 5:**
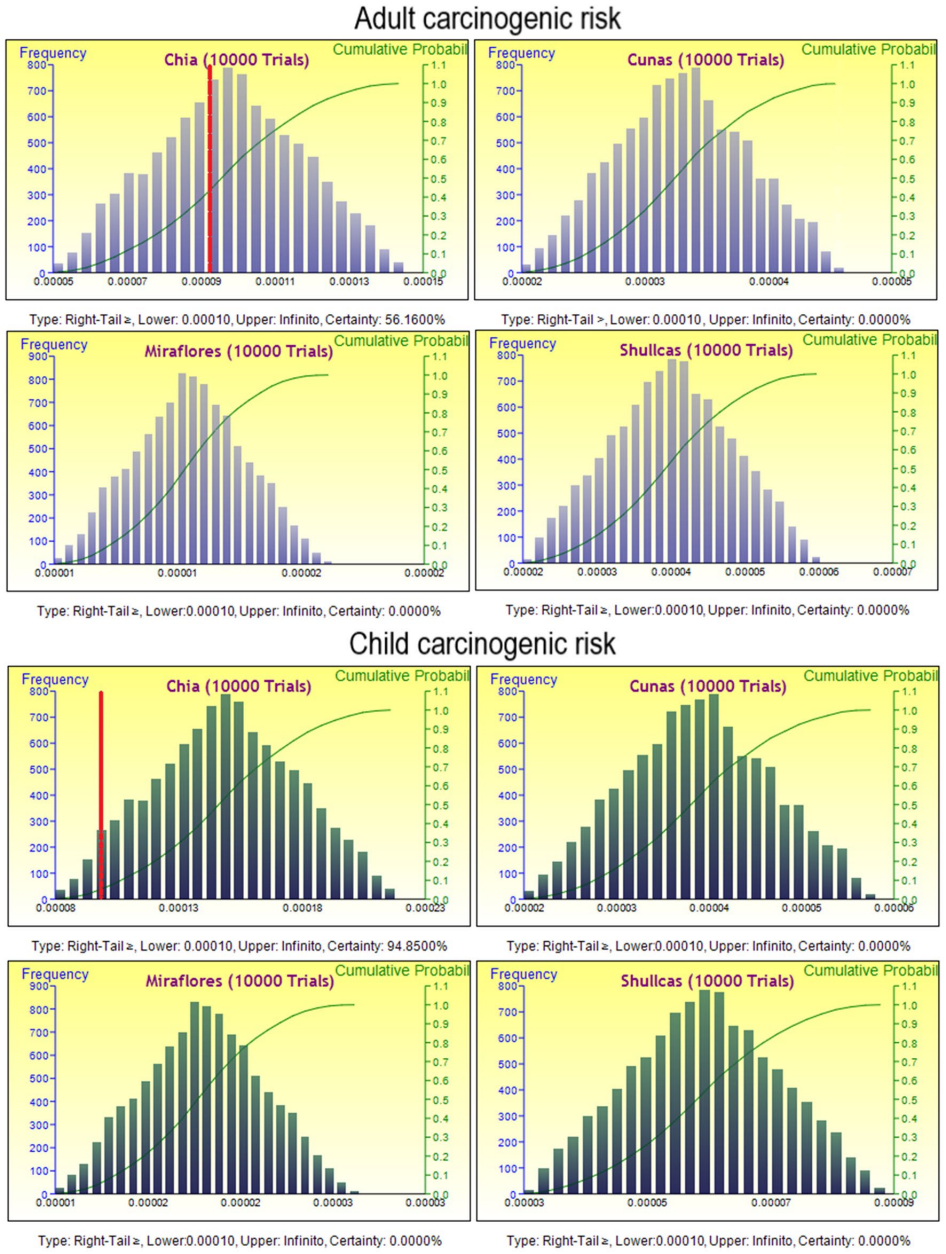
Simulated values for carcinogenic health risk in adult and child for consumption of water.

The results of the analysis of carcinogenic and non-carcinogenic risk for PTEs and arsenic in adults and children using a Monte Carlo simulation approach in the Chia, Cunas, Miraflores and Shullcas rivers indicate that the water of the Chia river for human consumption presents a high risk of contamination by PTEs, especially the carcinogenic risk for children. According to the simulation, there is a 56.16% probability of exceeding the limit value of 0.0001 for the carcinogenic risk in adults, while for children the probability value for the event of exceeding the value is 94.85% (Fig. 5). This is due to the high arsenic concentrations detected. These results are consistent with previous studies that have found that the presence of arsenic in water can pose a significant risk to human health, especially in areas where contamination is high, such as in some mining areas^80,81^.

**Figure 6:**
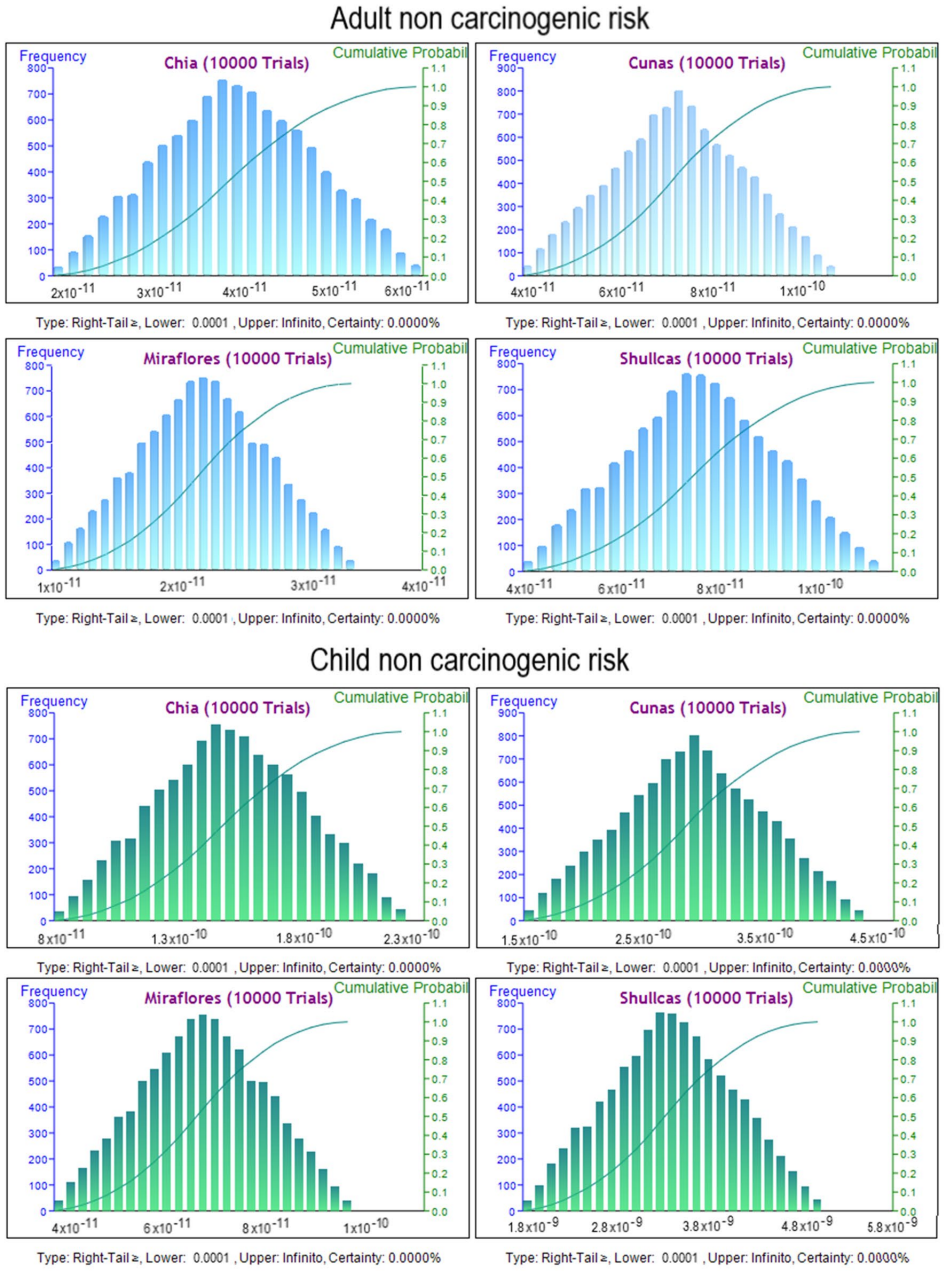
Simulated values for non-carcinogenic health risk in adult and child for consumption of water.

The results of the non-carcinogenic risk analysis for lead concentration in water, using a Monte Carlo simulation approach (Fig. 6), indicate that none of the rivers evaluated exceed the limit value of 0.0001, suggesting that lead exposure does not pose a significant risk to human health in these areas. However, the toxic effect of lead can cause a variety of health problems, such as insomnia, fatigue, hearing loss and weight loss, especially in children and pregnant women^82^. The results found in this study are in agreement with other studies that have found that the level of lead in water is low and does not pose a significant risk to human health^83^.

## Conclusions

Quantitative Microbial Risk Assessment (QMRA) is a tool widely used in community settings to predict the likelihood of infection and illness due to exposure to microbiologically unsafe water. The findings indicate widespread contamination of the four rivers by *E. coli*, *P. aeruginosa* and enterococci, with very high risks of disease, especially in the Chia river. The mean values of PTEs recorded in the rivers studied were lower than their corresponding water quality standard, except for As recorded in the Chia river. The levels of this metalloid may be of concern when considering long-term chronic exposure through water consumption or bioaccumulation and bioamplification in the food chain. The results also reveal moderate to strong positive correlations between physicochemical parameters and PTEs. The cancer risk analysis for heavy metals reveals that drinking water from these rivers has high probabilities of generating cancer, especially in the Chia river where the Monte Carlo simulation indicates a probability of exceeding the cancer limit of 56.16% in adults and 94.85% in children. Therefore, it is essential to take water pollution control measures to mitigate these significant risks to public health. Regarding bioremediation strategies, in our opinion, microbial bioremediation should be the preferred approach to control the high arsenic concentrations detected in the Chia river, given the genetic diversity and adaptation of microbes to the geographical conditions. While to reduce the occurrence of infectious agents in the waters of the rivers studied, the municipal governments of the districts in the study area should provide sanitation services to the population located along the rivers.

This work has been financed with EX-FEDU funds from the Universidad Nacional del Centro del Perú under Research Project No. 012022039753. We also express our gratitude to the Water Research Laboratory of UNCP.

### Supplementary Information

Supplementary Information.

## Data Availability

Data generated in this study are archived on Figshare.com via https://doi.org/10.6084/m9.figshare.23576397.v1. All other data relevant to this study are reported in the manuscript.
