# Supplementary material for: Microbial and potentially toxic elements risk assessment in high Andean river water based on Monte Carlo simulation, Peru
Source: Sci Rep. 2023 Dec 6;13:21473. doi: 10.1038/s41598-023-48853-4 (PMC10697974; doi:10.1038/s41598-023-48853-4)
Supplement: Supplementary file 1 — Supplementary Information. [file 41598_2023_48853_MOESM1_ESM.docx]

**Microbial and heavy metal risk assessment in high Andean river water based on Monte Carlo simulation, Peru**

**María Custodio^1^*, Richard Peñaloza^1^, Salomé Ochoa^1^, Heidi De la Cruz^2^, Ciro Rodríguez^1^, Walter Cuadrado^3^**

^1^Centro de Investigación en Medicina de Altura y Medio Ambiente, Facultad de Medicina Humana, Universidad Nacional del Centro del Perú, Av. Mariscal Castilla N° 3989-4089, Huancayo, Perú.

^2^ Facultad de Ingeniería Química, Universidad Nacional del Centro del Perú, Av. Mariscal Castilla N° 3989-4089, Huancayo, Perú.

^3^Universidad Nacional Autónoma Altoandina de Tarma, Jr. Huaraz 431, Tarma, Perú.

* Corresponding author: [mcustodio@uncp.edu.pe](mailto:mcustodio@uncp.edu.pe)

**Supplementary material**

| **Parameter** | **Abbreviation** | **Values** | | **Units** | **Reference** |
| --- | --- | --- | --- | --- | --- |
|  |  | **Adult** | **Children** |  |  |
| Element Concentration | $C_{water}$ | Measured | Measured | mg/L | Present Study |
| Daily Ingestion Rate | $IngR$ | 2.5 | 0.78 | L/d | ^1^ |
| Exposure Skin Area | $SA$ | 16,600 | 12,000 | cm^2^ | ^1^ |
| Conversion Factor | $CF$ | 0.001 | 0.001 | L/cm^3^ | ^2^ |
| Skin Permeability Coefficient | $PC$ | 0.0001 => (Pb)  0.001 => (As and Cd)  0.002 => (Cr) | | cm/h | ^1^ |
| Exposure Frequency | $EF$ | 350 | 350 | d/a | ^1^ |
| Exposure Duration | $ED$ | 70 (Cd, Cr and As)  35 (Pb) | | a | ^1^ |
| Exposure Time | $ET$ | 0.33 | 0.18 | h/d | ^2^ |
| Body Weight | $BW$ | 70 | 15 | kg | ^3^ |
| Average Time | $AT$ | 25,500 (Cd, Cr and As)  12,775 (Pb) | | d | ^1^ |
| Average Life | L | 70 | 70 | a |  |

**Table S1.** Value of health risk due to heavy metal exposure.

| **Non-carcinogen** | **RfD (mg kg^-1^ d^-1^)** | | **Reference** | **Carcinogen** | **SF (mg kg^-1^ d^-1^)** | | **Reference** |
| --- | --- | --- | --- | --- | --- | --- | --- |
|  | **Ingestion** | **Dermal** |  |  | **Ingestion** | **Dermal** |  |
| Pb | 1.4×10^-3^ | 4.2×10^-4^ | ^1^ | Cd | 6.1 | 0.38 | ^1^ |
|  | | | | Cr | 41 | 0.5 | ^1^ |
|  |  |  |  | As | 15 | 3.66 | ^1^ |

**Table S2**. The cancer slope factor (SF) and reference dose (RfD) of heavy metals.

| **Explanatory** | **Axis 1** | **Axis 2** |
| --- | --- | --- |
| Temperature | -0.33 | 0.24 |
| pH | 0.30 | 0.14 |
| OD | -0.23 | 0.10 |
| EC | -0.06 | 0.09 |
| TDS | 0.51 | -0.02 |
| TSS | 0.16 | -0.03 |
| Cadmium | 0.47 | -0.03 |
| Arsenic | -0.26 | 0.24 |
| Chrome | 0.29 | -0.07 |
| Lead | 0.59 | -0.03 |
| **Response** | **Axis 1** | **Axis 2** |
| *Escherichia coli* | -0.16 | 0.004 |
| *Pseudomonas aeruginosa* | -0.45 | 0.89 |
| Enterococos | -0.88 | -0.46 |
| **Eigenvalue** | **1.13** | **0.42** |
| **Proportion Explained** | **0.58** | **0.22** |
| **Cumulative Proportion** | **0.58** | **0.80** |

**Table S3**. Redundancy analysis (RDA) of physico-chemical parameters and heavy metals against the response of faecal indicator bacteria and pathogens.


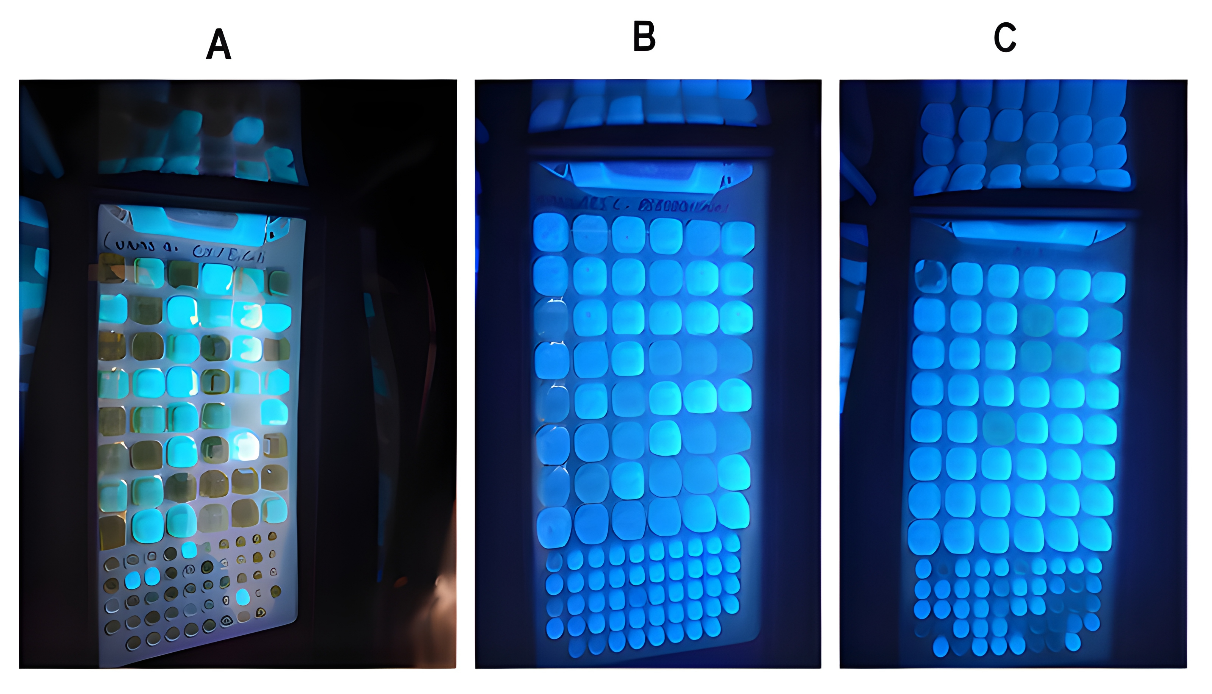


**Figure S1**. Indicator bacteria of faecal contamination and pathogens: A. Escherichia coli, B. Pseudomonas aeruginosa, C. Enterococos.

**Reference**

1. USEPA. Screening Levels (RSL) for chemical contaminants at superfund sites. (2011).

2. Jiang, C. *et al.* Distribution, source and health risk assessment based on the Monte Carlo method of heavy metals in shallow groundwater in an area affected by mining activities, China. *Ecotoxicol. Environ. Saf.* **224**, 112679 (2021).

3. Maleki, A. & Jari, H. Evaluation of drinking water quality and non-carcinogenic and carcinogenic risk assessment of heavy metals in rural areas of Kurdistan, Iran. *Environ. Technol. Innov.* **23**, 101668 (2021).
